# Addressing a need. Holistic midwifery in the Netherlands: A qualitative analysis

**DOI:** 10.1371/journal.pone.0220489

**Published:** 2019-07-30

**Authors:** Martine Hollander, Esteriek de Miranda, Frank Vandenbussche, Jeroen van Dillen, Lianne Holten

**Affiliations:** 1 Department of Obstetrics, Amalia Children’s Hospital, Radboud University Medical Center, Nijmegen, the Netherlands; 2 Department of Obstetrics, Amsterdam UMC, Academic Medical Center, Amsterdam, the Netherlands; 3 AVAG School of Midwifery and Amsterdam UMC, VU/EMGO Research Institute, Amsterdam, the Netherlands; Western Sydney University, AUSTRALIA

## Abstract

The Netherlands has a maternity care system with integrated midwifery care, including the option of home birth for low risk women. A small group of Dutch (holistic) midwives is willing to assist women in high risk pregnancies during a home birth against medical advice. We examined holistic midwives’ motivations and way of practice, in order to provide other maternity care professionals with insight into the way they work and to improve professional relationships between all care providers in the field. An exploratory qualitative research design with a constructivist approach and a grounded theory method were used. We performed in-depth interviews with twenty-four holistic midwives on their motivations for working outside their professional boundaries. Open, axial and selective coding of the interview data was done in order to generate themes. We held a focus group for a member check of the findings. Four main themes were found: 1) The regular system is failing women, 2) The relationship as basis for empowerment, 3) Delivering client centered care in the current system is demanding, and 4) Future directions. One core theme emerged that covered all other themes: Addressing a need. Holistic midwives explained that many of their clients had no other choice than to choose a home birth in a high risk pregnancy because they felt let down by the regular system of maternity care. Holistic midwives appear to deliver an important service. They provide continuity of care and succeed in establishing a relationship with their clients built on trust and mutual respect, truly putting their clients’ needs first. Some women feel let down by the regular system, and holistic midwives may be the last resort before those women choose to deliver unattended by any medical professional. Maternity care providers should consider working with holistic midwives in the interest of good patient care.

## Introduction

In the Netherlands, midwives can work in several settings and functions. The majority (72%) work as community midwives in primary care. The other 28% work as clinical midwives in hospitals, under supervision of an obstetrician [1]. Most of the community midwives work in group practices (80%), with an average size of four to six midwives who together provide care for approximately 90 women per midwife per year. They share antenatal check-ups and on calls, so their clients see most or all midwives in the practice in turn. Around 15% of community midwives practice as a duo and 5% work in a solo practice [1].

In recent years, case-load midwifery has made an appearance in the Netherlands. Case-load midwifery is defined as a (community) midwife who accepts a limited number of clients per month (four to five is average), for whom she is the only care provider. This means she personally performs all antenatal checks and also attends the birth herself, with a few exceptions for holidays or illnesses. In a duo or group of case-load midwives, midwives may take turns being on call for their combined clientele. Some of the hallmarks of case-load midwifery are continuity of care, a holistic (multidimensional) approach to pregnancy and childbirth, extra time spent per woman, and a personal relationship with the client [2]. Case-load midwives all deliver midwife-led continuity of care, which has been shown in a recent Cochrane review to result in less interventions during birth and a higher satisfaction in mothers [3].

In most developed countries, home births attended by midwives are relatively rare and are generally considered by most obstetricians as ‘against medical advice’ [4]. These days, most births in high income countries take place in hospitals, and if women refuse recommended care, midwives usually ‘side with’ the obstetricians in trying to persuade women to go along with the advice [5,6]. In contrast to the situation in the Netherlands, where home birth for low risk women is considered a regular choice within standard maternity care, in countries with a system where birth in itself is considered high risk, like the United States, home birth midwives may not distinguish between low and high risk births [7].

Community midwives in the Netherlands provide complete antenatal, natal and postnatal care to women with a low risk pregnancy. Women with a high risk pregnancy, or those who become so, are referred to hospitals for secondary (specialized) care, either during pregnancy, childbirth or postpartum, where they are attended by (trainee) obstetricians and/or clinical midwives working under an obstetrician. Half of the women who give birth in the care of a community midwife do so at home, the other half in a hospital or birthing center. Approximately 43% of women who started labour in the care of their community midwife are referred to secondary care during or immediately after childbirth [8]. However, there is a perception among maternity care providers that an increasing number of women refuse to be referred. They opt for a home birth in a high risk pregnancy with a midwife present. This ‘birthing outside the system’ includes, among other indications, post term pregnancies, twin pregnancies, breech births and vaginal births after previous caesarean section (VBAC). Midwives are not supposed to supervise these births at home, as this is in conflict with official guidelines and agreements between professionals. Therefore, the vast majority of community midwives does not want to take responsibility for those births. A small number of midwives (mostly working case-load) have stepped forward and are willing to assist women, who refuse the recommended hospital care, with a planned home birth. We will from here on refer to these midwives as “holistic”, since this is the term used most frequently in literature [9,10].

The appearance of holistic midwives in Dutch maternity care is a relatively new development. The first Dutch holistic midwife started her current way of practicing around the year 2000. It took several years for a second holistic midwife to join her, but by the beginning of the next decade there were more than a dozen, and current (2019) estimates range between 20 and 30 holistic midwives active in the Netherlands. In contrast, in 2016 there were 2315 practicing members of the Royal Dutch Organisation of Midwives [11]. The group of holistic midwives therefore represents about one percent of all working community midwives in the Netherlands.

In 2014, a highly publicized court case took place, in which the Health Inspection brought charges against three holistic midwives for assisting during home births involving twins and breech births [12]. After final review at appeal, the court recognized the fact that these midwives were the only assistance some women would accept during childbirth and that any assistance was better than none. They were therefore acquitted, although one was penalized for not making sufficient case notes to substantiate why she was in attendance and for performing inadequate resuscitation on a neonate. The court further stipulated that birthing women should not be left alone by their caregiver against their wishes, even if this means that the midwife has to attend a high risk birth at home.

Prompted by this court case, the WONDERstudy (Why women want Other or No DElivery caRe) was conceived in order to gain more insight in the phenomenon of women refusing recommended hospital care and opting for a home birth in a high risk pregnancy. The WONDERstudy is a mixed methods study examining women’s motivations to choose home birth in a high risk pregnancy or unassisted childbirth (UC), the motivations of their caregivers to assist them, and explores the magnitude of birthing ‘outside the system’ as perceived by midwives and gynaecologists, as well as their opinion on this phenomenon [13–16].

In a country known for its physiological approach to childbirth, relatively low intervention rates and high percentage of home births, it might seem strange that there is an apparent need for holistic midwives. In order to elucidate this matter, we interviewed the majority of holistic midwives in the Netherlands, who are willing to accept women with a high risk pregnancy for home birth, on their motivation to work the way they do, on how they feel about Dutch maternity care and on what distinguishes the way they practice from regular community midwives.

## Methods

We used the Coreq criteria for qualitative research to describe data analysis and report our findings [17]. We sought permission to perform this study from the medical ethics committees of the Radboud University Medical Center Nijmegen and the Academic Medical Center in Amsterdam, who both judged that the study did not require ethical approval.

### Research team and reflexivity

All interviews were conducted by either MH, EdM or LH, who are all women and professionals with a background in midwifery/obstetrics. All had experience with conducting interviews, and one (LH) had had previous interviewing experience during her PhD studies in medical anthropology. All interviewers had a professional interest in organization of maternity care in general and the phenomenon of home birth in high risk pregnancies specifically, and were known in the field as supporters of women’s rights and autonomy. Some of the participants were known to the interviewers through conferences, workshops, professional networks or social media, but only one participant had had professional contact with one of the interviewers (MH) through a shared case.

### Study design

This exploratory qualitative research used an interpretative approach to gain a better understanding of the phenomenon of holistic midwifery in the Netherlands. An interpretative approach focuses on interpretations of the world as it is subjectively understood, on the meanings that people give to certain phenomena, rather than on an objective reality. Following Charmaz, this study used an abbreviated constructivist grounded theory method and analysis [18]. A grounded theory method is used to develop theory from empirical data by first fragmenting the (interview) data into codes, the constant comparison of these codes, the grouping of codes into themes and finally by determining an overarching theme. A *constructivist* grounded theory method emphasizes the subjective interrelationship between the researcher and participant, and their co-construction of meaning. In this method the researchers are seen as “the author of a reconstruction of experience and meaning”, and as such their values must be acknowledged as an inevitable part of the outcome [19]. An *abbreviated* grounded theory method was used, as due to time constraints, only one cycle of data collection and analysis could be performed.

Participants were selected predominantly through a purposive sampling method: two of the midwives involved in the court cases mentioned above were approached. In addition, all Dutch midwives known to the researchers to be practicing in a holistic manner and known to be prepared to assist women with home birth in a high risk pregnancy were asked to participate. Finally, a snowball method was used, by which all participants were asked if they knew of any colleagues working similarly. These were then also requested to participate. All participants were approached by either e-mail or private message on a social media platform. In total, 28 midwives were approached, which, according to those interviewed, represent the vast majority of holistic midwives working in the Netherlands at the time. Four midwives refused to participate. Reasons for refusal were cited as “Uncertainty about the agenda of the study group,” “Not trusting confidentiality” and “Lack of time”.

All interviews took place in a location chosen by the participants, which was most often either in their home or place of work and rarely in a public place like a café. One interview was done by telephone, due to logistical reasons. All conversations took place in private. Data were collected between the summer of 2014 and the spring of 2016 and all participants gave verbal consent for their quotes to be used in this article.

**Fig 1:**
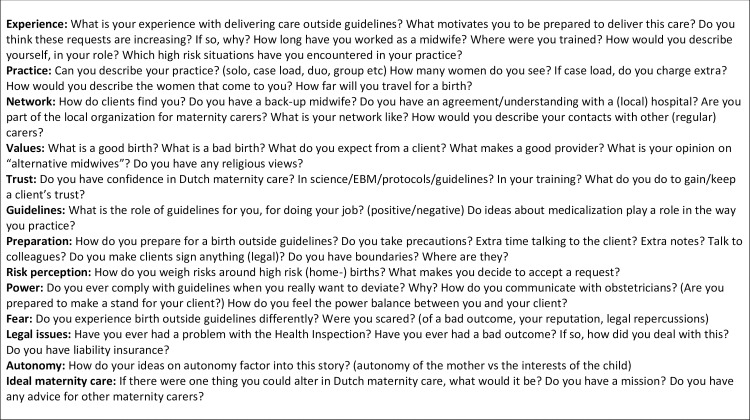
Topic list.

The interviews were semi-structured by use of a topic list (Fig 1), which was based on questions the researchers had after studying the literature at the start of the study, and this was adjusted throughout the study as new themes were brought forward by the participants. The interviews were allowed to take a spontaneous course and lasted between 45 and 150 minutes. Most midwives were interviewed once, with two having two separate sessions. Interviews were recorded by digital sound recorder and transcribed verbatim by either a commercial company or volunteer medical students. All sound files and transcripts were stored anonymously in a secured password protected university digital storage system. In September of 2017, a feedback focus group was held with six of the participants, four of whom were physically present, and two were on speakerphone.

The first author (MH) translated all quotes into English.

### Data analysis

**Fig 2:**
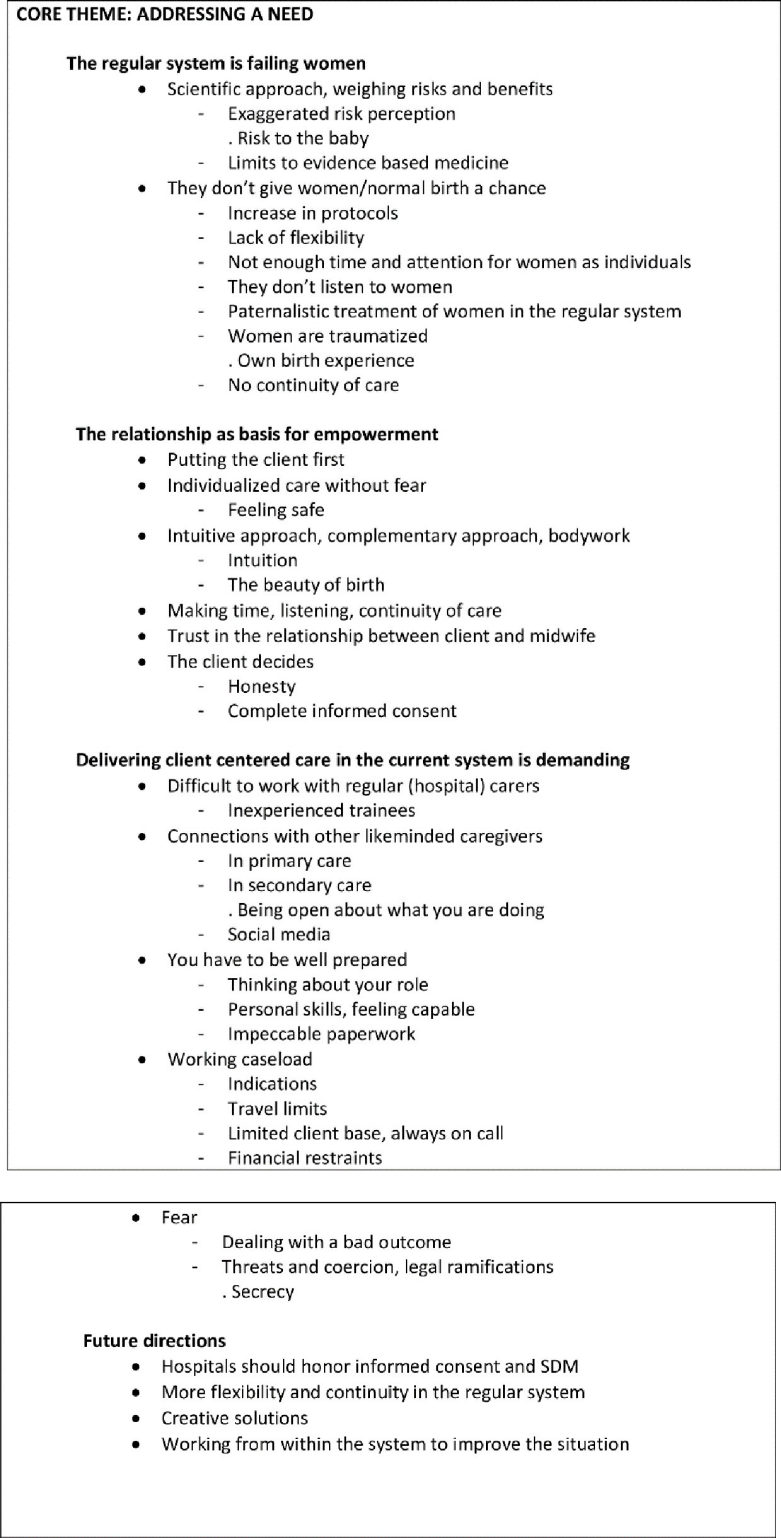
Coding tree.

The first author (MH) analyzed the data, with LH coding one interview halfway through the process to check for any missing codes. We used qualitative data analysis software program MaxQDA (VERBI GmbH) for the coding process. Open coding was started from the bottom up, with codes being added and expanded on as new interviews were coded. The sensitizing concepts of shared decision making and continuity of care were used as a lens in coding the interview data. We grouped codes in themes and subthemes, after which a core category emerged. Data saturation was reached after 21 interviews, with the final three interviews being coded to confirm this. The codes were grouped into the final coding tree (Fig 2), which LH and MH decided on by consensus after all coding had been completed.

## Results

Twenty-four midwives were interviewed. The majority (66.6%) of participants practiced case-load midwifery, sometimes as a solo practitioner, sometimes in a group or duo (Table 1). Three midwives worked in a regular group practice, in which clients were shared. These individual midwives were willing to assist women who refuse the recommended hospital care with a planned home birth. The average age of participants was between 40 and 50 years old, and they had been practicing midwifery for an average of fourteen years, with a range of two to thirty years. The first participant who started a holistic case-load practice did so around the year 2000, and most have been working this way for less than ten years. Fourteen midwives (58.3%) worked in an urban setting.

**Table 1:** Characteristics of the participants.

Age	21–30	1 (4.2%)
	31–40	9 (37.5%)
	41–50	10 (41.7%)
	51–60	4 (16.7%)
Work experience	0–10 years	9 (37.5%)
	11–20 years	10 (41.7%)
	21–30 years	5 (20.8%)
Average	14.2 years	
Practice	Urban	14 (58.3%)
	Semi-rural	4 (16.7%)
	Rural	6 (25.0%)
Work setting	Solo	
	- Case load	12 (50.0%)
	- Non case load	2 (8.3%)
	Duo	
	- Case load	1 (4.2%)
	- Non case load	2 (8.3%)
	Group	
	- Case load	3 (12.5%)
	- Non case load	3 (12.5%)
	Locum	1 (4.2%)

After grounded theory analysis of all interviews, four major themes emerged: “The regular system is failing women”, “The relationship as basis for empowerment”, “Delivering client centered care in the current system is demanding” and “Future directions”. After careful consideration of all data, it became clear that one overarching core theme connected all other themes and subthemes. This core theme was “Addressing a need”.

Six participants took part in the feedback focus group. All agreed that the results were a fair representation of their stories; however, they all felt that there needed to be more emphasis on their physiological approach to childbirth. We incorporated these remarks in the emphasis we have placed on certain themes. While organizing the feedback focus group 18 months to three years after the interviews, we reconnected with as many participants as we could and found that nine of the 24 were no longer in practice, or at least not as holistic midwives. Of the fifteen midwives who were still active in their holistic practice, two were currently trying to decide if they want to keep working in that setting.

### The regular system is failing women

An important motivation for participants to accept women with a high risk pregnancy for home birth is their conviction that the regular maternity care system in the Netherlands is failing women. They felt that there is too much emphasis on risk talk and not enough room for a physiological approach to childbirth. Participants believed that the regular system relies too much on protocols, at the expense of patient centered care, and lacks time, attention, and continuity. They stressed that frequent use of a paternalistic approach and lack of informed consent traumatize women.

All participants described the current biomedical discourse and reliance on protocolized care as too dominant. They pointed out that, although evidence based medicine (EBM) is an improvement to authority based medicine, it is often used in too limited a sense. One of the cornerstones of EBM is patient preference, which they felt is not taken into account often enough, in the presence of protocols which provide strong recommendations:

*“It is everywhere in all the guidelines (…)*: *the client comes first*. *I don’t think the client comes first*, *I think the guidelines come first*. *And they are no longer guidelines*, *they have become laws*.*”* (Midwife 20)

The participants felt that due to the increase of protocols recommending interventions, women have less options to have an intervention-free birth:

*“Because I wouldn’t want to be there*, *in that setting*. *With everything they have*, *you know… (…) one is ready with the syringe*, *the other with the vacuum*, *the third with an iv*. *(…) That is war*, *instead of cooperating with the forces of nature and the woman’s power*.*”* (Midwife 6)

Participants also described maternity care providers’ (both doctors’ and midwives’) fear of litigation tends to make them overly cautious, whereby they would rather do too much than too little:

*“I believe that maternity care as it is now is going in the wrong direction*, *because I feel like… (colleague) said it very well*: *‘People would rather be judged for the things they did than for the things they didn’t do’*, *that we are almost headed for an American system*, *that we all try to rule out as many risks as possible (…) with the idea*: *at least we tried everything*.*”* (Midwife 21)

When asked why women approached them, most participants stated that the current maternity care system does not give women an honest chance of a normal birth.

*“It’s just like*: *this is how it’s done*, *this is how the infusor is adjusted*, *this is how the CTG is done and this is how long you are allowed to push and you should do it in such and such a way… So it becomes*, *yes*, *it becomes almost a little bit like a factory*.*”* (Midwife 9)

Participants also complained about lack of time and continuity in regular maternity care. Women are confronted with many different providers, who have very little time to spend per consultation.

*“Colleagues (regular midwives) would say*: *‘You give patients so much time*, *soon they will want the same from us*, *we can’t do that*, *we don’t have the time for it*!*’ So you are just in a (group) practice where you see six people in an hour and when someone says*: *‘I am so afraid of giving birth*!*’ then you say*: *‘I have three minutes to talk about it’*, *or*, *‘Don’t worry about it*. *You have us*! *You will be fine*!*’”* (Midwife 21)

Participants reported that one of the most important reasons women come to them is because many have been traumatized in regular care, often due to loss of autonomy.

*“When the woman was not heard*, *when decisions have been made without consulting her*, *(…) when doctors come in and…*.*when they go like this with two fingers and immediately go for your vagina instead of shaking your hand first*, *you know*, *I find that really excruciating*.*”* (Midwife 21)

According to many participants, maternity care is still a very paternalistic field, in which women are frequently coerced and informed consent is optional. When women refuse certain parts of the protocol, there is no flexibility and no concessions are made, leading the women to take extra risks by approaching holistic midwives for home birth assistance in high risk pregnancies:

*“And there was a lady who would have been willing to mostly conform to the guidelines*, *but the door was slammed in her face*. *I think based on the conviction*, *that if you just keep saying no*, *then…*.. *Like with a child*, *if we just keep saying no*, *at some point they will accept it (that they have to follow protocol)”* (Midwife 7)

*“For instance intermittent auscultation in a vaginal birth after Cesarean*, *instead of continuous monitoring*. *That was (…) not done*. *I dragged her to six hospitals in our area*, *and everywhere we were told no*. *Until the lady said*: *‘Then I will stick with my decision*, *I will birth at home*.*’”* (Midwife 18)

### The relationship as basis for empowerment

The midwives who participated in this study have a different and personal approach to maternity care compared to the regular system. Building a trusting relationship with their clients is at the core of their work. This means that there has to be a personal connection between midwife and client, which serves to empower the client to take responsibility for her own process:

*“If a client approaches me the first thing I ask myself (about her) is*: *can you carry yourself*? *Are you (…) prepared to deal with your process and reflect on it*? *And are we capable of establishing a meaningful relationship*?*”* (Midwife 8)

Holistic midwives want to deliver individualized care without fear. They believe decisions in maternity care should be made based on the individual woman, her experiences and her preferences, not taken directly from any guideline or study. They may treat the guidelines as a starting point for negotiations, but some discuss all possible options, including those not recommended in the guidelines:

*“Yes*. *I go along with every wish*. *If I feel like people are actually taking responsibility for it themselves*. *I have to feel like they have thought it through sufficiently*. *That they are coming from a position of strength and not from fear*. *For instance*, *not from fear of ending up in a hospital*, *but from a conviction or a trust in something*: *in themselves*, *or whatever*. *I have to feel like they can carry themselves*. *If not*, *I won’t do it*.*”* (Midwife 4)

Participants believed that honesty and trust in the relationship are prerequisites for going outside the boundaries of the protocols:

*“The rest is all about talking to each other to determine if we can trust each other*. *(…) That is what the whole care is about*. *If there was something not right or something didn’t fit*, *that would be a reason for me to evaluate*: *am I still the right caregiver for you*?*”* (Midwife 17)

Some women are so set against going to the hospital that they are almost impossible to persuade to be referred. However, the majority of participants found willingness to be referred an important condition for their partnership with their clients. If the midwife feels that that trust is lacking, she may not be prepared to go forward in her role as caregiver:

*“When we get to this point*, *that means there is something in our mutual confidence that is not right*. *At first I failed to get the conversation to that level*. *I thought*: *I don’t want it this way*. *First the foundation has to be right*, *then we can move on*.*”* (Midwife 24)

Long consultations, listening to women’s needs and continuity of care are the hallmark of case-load midwifery, as practiced by the majority of the participants. They frequently see their clients for an hour or more each time, and they are usually the only midwife their clients see. This builds the trust necessary for clients to be willing to follow the midwife’s advice in case of a need for referral during the birth:

*“Probably because you see each other again and again (…) women say*: *‘If you say that there is a limit*, *I will completely follow you*.*’ They dare to put it in your hands*, *while before they were afraid to put it in anyone’s hands*, *because you have spoken a lot and because they know I take them seriously*.*”* (Midwife 14)

Some participants who worked in a regular group practice experienced a lack of opportunity to build such trust with the majority of their clients, since they did not have enough time to get to know the them:

*“I have noticed*, *for instance*, *now that we are four (midwives)*, *some women I see once*, *twice in the pregnancy*. *I don’t build a relationship with those anymore*.*”* (Midwife 22)

The majority of participants believed that going outside protocol should always be at the client’s suggestion, and the client should always have the final say in every decision:

*“What do I do in my job*? *I listen to what people want*. *And it is nice that we have standards*, *guidelines*, *rules and what not*, *but the most important thing is*: *what does the woman want*?*”* (Midwife 11)

However, not all participants counsel their clients with complete neutrality on all options. Some do allow their own opinions to occasionally guide their advice to their clients, or steer them in a certain direction. In some cases that may lead the midwife to perform less check-ups and interventions than are recommended, because of her personal opinion about these interventions:

*“If someone is birthing in the bath*, *in a natural position*, *and is pushing without being coached*, *I don’t find it necessary to listen (to the heartbeat) quite so often*, *and I determine that for that woman*.*”* (Midwife 15)

A minority of participants had a more intuitive approach to their work. Some practice complementary techniques such as homeopathy, herbal remedies and massaging techniques. During their work they are often guided by their own as well as the mother’s intuition in judging fetal condition or labor progression. They see birth not as a medical occurrence, but as something the female body was made for:

*“That decision that I want to follow my heart has been a very important choice*. *My heart was my guideline*, *you see*, *not the protocol or the rules*, *but just my heart and my intuition*.*”* (Midwife 6)

### Delivering client-centered care in the current system is demanding

Being prepared to accept women with a high risk pregnancy for home birth and working (mostly) case-load is often rewarding for these midwives, but frequently also a heavy burden. Participants feel stigmatized or even villified by regular maternity care providers, and they are made to fear legal repercussions in case of a bad outcome, which makes working the way they do emotionally difficult. They spend much time preparing and planning for a particular birth, and most try to be open about what they are doing. Most participants are almost always available for their clients, for little financial reward, and sometimes have to travel far outside their own region.

One of the things that participants have the most difficulty with is their working relationship with regular providers, sometimes community midwives, mostly obstetricians (and trainees) in hospitals:

*“But there are also midwifery practices who don’t want to be (my) back-up and then I just don’t have…a back-up*. *Because then no-one in the whole area wants to be (my) back-up and that is the end of it*. *Then it is just a longer drive*. *So what I discuss with the client is that the hospital is basically the back-up at such times and that in case of an acute emergency she should just…call the ambulance*. *That*, *in my opinion*, *does not constitute the best care*, *but it is what it is*.*”* (Midwife 4)

*“In the eleven years I have worked I have been told off by an obstetrician and I have been ignored many times*. *There are obstetricians who still ignore me*. *I always find that very intimidating and I let myself be intimidated*.*”* (Midwife 15)

However, not all contact with hospital care has been disrespectful, as one participant discussed:

*“‘Well*, *(name midwife)*, *you have so much experience*, *when YOU send someone to us it must really be necessary*.*’ (…) If you come they know*: *yes*, *it is necessary*, *you have already tried everything*, *so we are not going to try that again*. *And yes*, *to me that is a basis of equality*.*”* (Midwife 20)

Several of the participants reported explicitly that they attach much value to being transparent about what they are doing. This means that when they have a client who wants to go outside guidelines and have a home birth in a high risk pregnancy, they attempt to persuade her to go for at least one consultation in the local hospital. That way, the client is already known there, in case of the need for an urgent referral, and less eyebrows will be raised when she shows up during labor. The holistic midwives will usually accompany their clients during such a consultation:

*“Because I don’t want to be secretive*. *I explain that to people*, *too*: *I won’t do that*. *We are going to the hospital together and we will have a conversation there*. *You can explain why you want this*. *Just so they know*, *and I also want there to be a record of it*, *so that we are welcome in case of a complication*.*”* (Midwife 5)

In spite of this transparency, many participants reported fear of legal repercussions. They are frequently threatened by regular caregivers with reports to the Health Inspection. Since the 2014 decision by the court cited previously there have been no more convictions; however, several cases have been investigated:

*“And that obstetrician has threatened that*, *actually*. *(…) Like*: *‘I think we are going to have to report this*. *It is not right*, *and when you are facing the Disciplinary Board*, *then…’ Like that*.*”* (Midwife 5)

*“And she (community midwife) threatened me that if I would not stick to the protocol that she would go public and publish it in the newspapers and that sort of thing*.*”* (Midwife 6)

This has led one of the participants to avoid the openness avowed by the majority and try to keep her work outside guidelines out of the public eye. However, most participants are not fazed by threats:

*“I don’t want to be so afraid of such an institution (Health Inspection) that I can’t serve my women anymore*, *actually*. *That is what it comes down to*.*”* (Midwife 19)

To share the burden of working the way they do, most of the participants are part of a network of likeminded caregivers. They have a private facebook page they use to communicate, discuss cases and support each other in case of trouble or a bad outcome. They are also often back-up to each other, which means they will attend each others’ clients in case of holidays or illness. Some also have connections in a (local) hospital, where they know they can go if they need help or want to discuss a case:

*“This was a very kind doctor*. *I dropped her name in our little club*, *like*: *‘If you want something different*, *some time*, *you can try it there*.*’ (…) I was very content with that*.*”* (Midwife 13)

Working on a case-load basis can be very demanding. Due to the fact that there are very few midwives who will accept births outside guidelines, participants sometimes have to travel significant distances to reach their laboring clients. Some mentioned a limit of 45 minutes to an hour, while others said that if the need is urgent there is no limit to how far they will travel. In addition, few clients means little income, although a number of participants supplement this by asking extra fees from clients. As case-load midwives, they are always on call:

*“I almost never have a holiday*, *that is the downside*. *So I am really almost always available*. *(…) Yes*, *I always have my telephone… Always*, *yes*, *yes*. *I don’t know if the others have told you this*, *but yes*, *we really have a kind of phobia with that telephone*. *That telephone is always there*, *so you always know where your telephone is*. *That is the downside of working like this*.*”* (Midwife 9)

When working with a client who wishes to deviate from guidelines, holistic midwives spend extra time on preparation: going over their clients wishes, checking their own suitability and skills, and making extensive case notes. For instance, if their client will not allow them to monitor the baby and refer to the hospital if necessary, they try to ascertain what it is their client does want from them:

*“You know*, *if people are not willing to be referred*, *then I wonder what they want with me*? *What am I doing there*?*”* (Midwife 19)

In addition, the participants spend a lot of time meticulously documenting all conversations with their clients, both before and during the birth, since that documentation will be their only line of defense in case of a possible case brought by the Health Inspection:

*“So I wrote down*: *‘I have offered to listen to the heartbeat*. *She refused*.*’* (Midwife 13)

Two of the participants had experienced a bad outcome. This has not changed their practice; however, it has been a significant burden on their minds:

*“And then it is a rollercoaster you end up in*. *(…) The Health Inspection*, *and…*.*opinions of others and support of others*. *Sleepless nights…”* (Midwife 3)

Although all participants were convinced that their clients do not take decisions regarding their baby’s safety lightly, some still felt the burden of responsibility when clients appear to make decisions that pose increased risk to the baby:

*“(*..*) I find that*, *if a mother chooses that*, *she knows very well what she is choosing*, *but a baby doesn’t know what it chooses*. *(…) It doesn’t choose*. *So it doesn’t choose to be born breech at home*, *or it doesn’t choose to be a twin home birth*. *The parents choose that and therefore I find that more difficult than when someone with a previous post partum hemorrhage wants to stay home*, *or with heparin*, *if they have a clotting disorder*. *That to me is self determination over your own body*.*”* (Midwife 17)

### Future directions

After discussing their motivations for working the way they do and explaining the difficulties they sometimes face, the participants were asked what they would like to change about maternity care, if they could. Several participants suggested that hospitals and community midwives should start by implementing true informed consent and shared decision making in every consultation:

*“If there was one thing (I could change)*, *then it would actually be*: *try your best then*, *to get to know someone*. *And what motivates someone*, *and then often it won’t even be so unreasonable*.*”* (Midwife 7)

Participants also called for more flexibility and continuity of care in the regular system. Several mentioned as an example an integrated care protocol of one of the Dutch university hospitals, in which primary care midwives can stay with and assist their clients during a VBAC, as long as hospital protocol (continuous monitoring) is followed. Several of the participants came up with creative solutions to improve maternity care: for instance, obstetricians making house calls, or starting a chain of dedicated breech clinics:

*“I would really like a sort of breech center*. *Somewhere in the country*, *where there is a bath*, *and a midwife*, *and a woman-friendly obstetrician*, *who says*: *‘Come on*, *we’ll let this woman give birth the way she wants to give birth*.*’ To actually study that*, *and publish results*. (Midwife 1)

Finally, most of the participants reflected that they did not want to completely separate themselves from regular maternity care. They wanted to stay in the system, teach others and work from the inside to improve the situation for their women:

*“Some things have been set in motion*, *I think*. *I really do think those (integrated care) pathways have come about because we showed up quite a lot with people with these sorts of wishes*, *that we now have those pathways for*. *So there is a point to it after all*. *And I am going to keep doing it in the interest of caring for my women*.*”* (Midwife 13)

### Core theme: Addressing a need

The one pervasive theme that emanates from all interviews is that all participants felt the responsibility to be there for women who have, in the women’s own view, nowhere else to go. These midwives believed that there is a need for their services, since regular maternity care in the Netherlands is letting women down by not leaving enough room for physiological birth and not providing enough time, continuity, flexibility and respect for autonomy:

*“And then*, *for me*, *it became an activist kind of thing*, *like*: *this is very important*, *that we take a stand for this (…)*, *that women are entitled to their bodily integrity at all times*, *and can refuse care*, *but in the meantime should not be denied care*.*”* (Midwife 12)

The participants in this study claimed that they are meeting women’s needs by establishing a relationship built on trust, empowerment and mutual respect; by taking the time to get to know their clients, in order to serve them better; and by being available as a last resort for these women.

*“And so in some way I was the final destination*, *because after me there was nobody else who could say*: *oh well*, *go to the next midwife*, *she will do it*, *she will help you*. *(…) Yes*, *someone had to be with that woman*, *somebody had to do it*, *you know*.*”* (Midwife 6)

These midwives truly address the need for client centered care and also hope, by suggesting future directions, that regular maternity care will follow their lead. This sometimes comes at considerable cost to the midwife herself, both in her relationships with other care providers and in her personal life.

*“And that is why I feel it is a bit unfair*, *if an obstetrician says*: *‘The way you do it*, *you create this demand*.*’ I am only trying to facilitate what is already there*.*”* (Midwife 13)

## Discussion

Holistic midwifery is a relatively new phenomenon in the Netherlands. For this qualitative study into the motivations and practices of Dutch holistic midwives, we interviewed 24 midwives. Four main themes were found, which all led back to one core theme: addressing a need. Despite the fact that the Netherlands has an integrated home birth system and relatively low intervention rates compared to other developed countries, these midwives feel very strongly that there is a need among Dutch pregnant women that is not being met in the current maternity care system. We will now discuss this need and its’ effect on the midwives involved and look at implications for practice.

### Identifying the need: what is lacking in current maternity care?

#### The need for a physiological approach

Midwives in this study firmly believe in trusting the birthing body to know what to do. They stated that risk is often being introduced by unnecessary interventions and frequently mentioned a lack of room for physiological childbirth as the reason women gave for leaving the system and turning to them for assistance. Their impressions are supported by the changing statistics of Dutch maternity care. Currently, about 87% of Dutch women start their pregnancy in a community midwifery practice, but only about one in three deliver in primary care with their community midwife, either at home or in a hospital or birth centre [8]. In recent decades, the percentage of Dutch women being referred for secondary care at some point during pregnancy, birth or postpartum, has more than doubled, from 24.7% in 1964 to 58.3% in 2015 [8,20]. This is due to both an increase in (perception of) pathology, and the demands for hospital care and/or pain relief by the women themselves [21] as well as an increase in protocollized care. In addition, interventions during birth have risen steadily over the years, both in primary and secondary care [8,22]. For instance, in 2009, 17.7% of all births were inductions of labour, and 6.7% were planned caesarean sections, versus 22.6% and 8.0% respectively in 2016. Also, the percentage of epidural analgesia during the first stage rose from 13.9% in 2009 to 21.8% in 2016. However, as the WHO recommends in its recent guidelines on “Intrapartum care for a positive childbirth experience,” more patience in labour and less interventions lead to more positive experiences for women in childbirth [23]. The midwives in this study firmly agreed with this sentiment, linking the increase in interventions to the increase in traumatic birth experiences. There is strong evidence in favour of a more physiological approach to labour and birth than is currently being practiced, which is summarized in a consensus statement by US midwifery organizations [24]. The lack of room for a physiological approach to childbirth in regular maternity care reported by the midwives in this study is also a well-recognized phenomenon in other developed countries in recent years. In the UK, for instance, Scamel and Alaszewski point out that “In midwifery conversation, normality has no language of its own and has to be defined against the dominant discourse of high risk” [25]. The same lack of a physiological approach has been reported in Ireland, where Healy [26] describes how the obstetricians’ views on risk have increasingly prevailed over those of the midwife, whereby even low risk women are routinely required to labor on a continuous fetal monitor.

The participants in this study frequently mentioned that, when confronted with a woman who has a birth plan that is not in line with recommendations, medical professionals referred to guidelines and protocols and ‘evidence-based medicine’ as grounds for being unwilling to accommodate her wishes. However, evidence-based medicine has three pillars: rigorous scientific evidence, which is used to underpin protocols and guidelines, but also providers’ experiences, and, notably, patient preference [27]. According to the participants in this study, this focus on guidelines as the sole important component of EBM has led to a lack of flexibility and medicalisation in regular care. This is substantiated by a recent study among Dutch women who had a home birth in a high risk pregnancy or an unassisted childbirth in the Netherlands [15]. Many women in that study did not necessarily want a home birth, but found no room in the hospital to deviate even a little bit from standard protocol. The midwives in this study fulfill the need these women have for a physiological approach and flexibility in the use of protocols and guidelines.

#### The relationship as basis for empowerment

Participants in this study emphasized in interviews that one of the most important characteristics that sets them aside from other community midwifery practices and empowers their clients is the personal relationship holistic midwives establish with their clients. Holistic midwives always stay with their clients, regardless of transfers or handovers. The majority of women in the Netherlands currently encounter both community midwives and hospital staff (obstetricians, trainees and clinical midwives) during pregnancy and/or childbirth. As Offerhaus states: “The persisting rise in referrals challenges the sustainability of the current strict role division between primary and secondary maternity care in the Netherlands” [28]. When a woman is transferred during labor, it is usual for the community midwife to leave at some time after hand-over, unless the birth is imminent. Women are then left in the care of a new team whom they have never met, at a time when things are obviously not going according to plan and they are at increased risk of needing an intervention as well. They have had no time to build a relationship of trust with this team. This may well be an important factor in the reason why women blame their traumatic experience on factors of communication, support and explanation [29]. The participants in this study believe that the personal relationship they have with their clients is crucial. In medical literature, there has been some debate over whether the continuity that women are missing is continuity of care or continuity of caregiver. There are some studies which indicate that women do not mind being handed over to a new team, as long as there is continuity of care and information, and the policy agreed on in the birth plan is still followed [30]. However, there are growing indications that continuity of caregiver is actually very important for women’s satisfaction with childbirth [3,31–33]. A recent Dutch study emphasized the importance of continuity; they found that transfer of care and lack of continuity led to problematic communication and dissatisfaction on the part of the women [34]. Another study showed that, in the Netherlands, continuity of care as experienced by women is significantly higher in a midwife-led care model [29]. Davison et al. also found that for Australian women to be able to trust their providers, they need to be able to build a personal relationship with them [35]. This, participants have indicated, is becoming increasingly difficult; where in 1980 in the Netherlands, more than two-thirds of community midwives worked as solo practitioners and 8.8% in a group of three or more, by the year 2015 this had become the opposite, with only 5% of midwives working solo and 80% in a group of three or more [1]. In addition, group practices have been growing in size, with groups of six to eight midwives being common in recent years. The same phenomenon has occurred in hospitals, where, due to several factors such as feminization of the profession, increase in part-time work and mergers between different hospitals, women are now likely to see obstetrical groups of not three to five obstetricians, but frequently between ten and twenty [1]. In 1980, there were 7.5 maternity care providers per 1000 pregnant Dutch women, whereas in the year 2015, this had grown to 24.3 [1,11,36]. Therefore, the number of representatives of both professions that women encounter has more than tripled in the past four decades.

Participants in this study also indicated that women turn to them because they know they can trust that their birth plan will be respected, which is in line with previous reports [37]. As Perry et al. put it: “Lots of discussion beforehand and carefully negotiated treaties don’t turn out to be altogether reliable” [38]. In addition, as Sandall found, for some women it may be difficult to negotiate a birth plan with a different provider at each visit [39].

In summary, the average Dutch pregnant woman is likely to see many more different caregivers during her pregnancy and peripartum than several decades ago. Women are confronted with a large number of different caregivers with whom they have no opportunity to build a trusting relationship, which, according to the participants in this study, is the foundation of and a prerequisite for client centered care. The holistic midwives in this study meet that need by providing continuity and mutual trust.

#### A heavy burden

Addressing women’s need for a last resort takes a heavy toll on holistic midwives. There are currently less than thirty midwives in the Netherlands working in this manner. All participants in this study, at the time, expressed great love for their job and a motivation to go forward. They believed that their work was vital in order to be able to offer women the care they needed and couldn’t find in the regular maternity care system. Just as reported by Symon et al [40], who interviewed independent midwives in the UK, they felt that in many cases, if it had not been for them, women would have felt no other option than to give birth at home unassisted.

However, participants also voiced several drawbacks to being a holistic midwife in the current system of maternity care. Most importantly, participants felt a great deal of pressure by regular caregivers to stop attending home births in high risk pregnancies. Among other things, they were often accused of creating the demand they were facilitating, since several holistic midwives refer to care options outside protocol on their professional websites or in blog posts [41–43]. Some were also threatened with reports to the Health Inspection. Even though the initial three midwives in the court case referenced earlier had been cleared of all wrongdoing, many of the participants still felt anxious. They felt the pressure of constantly being judged by regular care providers, whose hostility, distrust and animosity weighed heavily on their minds. This is in accordance with the findings of Jefford and Jomeen [44], who report that independent midwives in the UK spoke of feeling ‘out on a limb’ and of ‘being blamed’ for women’s refusals, and of Symon et al. [40], who found that hospitals did not respect holistic midwives’ expertise. This theme of difficulty working with other providers in the chain of maternity care can also be found in the work of Kensington et al [45] and Crowther and Smythe [46] among midwives in rural practices in New Zealand and Scotland. In addition, as Crowther et al. found, in case of a bad outcome, midwives can fear the notoriety that may accompany being identified as the midwife who ‘was responsible for’ a dead baby [47]. Medico-legal tensions and fear for a bad outcome were also themes found in a recent meta-ethnography by Feeley on midwives who care for women making unconventional birth choices in the UK [48].

Another burden mentioned by those participants who worked case-load was the constant availability. They always had to be ready to leave at a moment’s notice, which meant that for those who had children, child care always had to be available too. This is again in accordance with the findings by Crowther et al., who also mentioned case-load midwives’ need to sometimes be away from the telephone [47].

Since participating in this study, nine of the 24 participants have changed their practice and are no longer working in a holistic manner. When we asked these participants why they gave up their practice, the most frequent reasons were the pressure exerted by regular care providers and the threat of legal action being taken against them. Another often mentioned reason was the burden of constant availability. Other reasons were lack of support by their professional organization and impossibility of meeting the insurance companies’ criteria for reimbursement. Although none of those interviewed mentioned the word, the motivations of these nine midwives to give up their holistic practice appear related to the phenomenon of burnout. As elucidated in the thesis of Young [49], “the phenomenon of burnout is little understood within midwifery, yet it seems that the nature of on call practice has a high potential for burnout”. As Sandall found in her 1997 study [50], even though high levels of occupational autonomy were a key protective factor of burnout, there have been growing concerns to suggest that the community-based continuity of care model may not be sustainable due to the high levels of occupational burnout in midwives resulted by increased on-call work. It appears that holistic midwives may be at greater risk of burnout exactly because they care so much about their work, since, as Lynch [51] suggests, “burnout happens most frequently to people who are passionate, idealistic, and who have chosen caring for others as their work”.

None of the midwives who had quit her practice had done so over a bad outcome. In spite of the heavy burden, five new midwives have started holistic case-load practices in the last three years, filling the gaps left by those who quit.

In summary, addressing a need is a heavy burden for many holistic midwives, due to pressure by other care providers, fear for their reputation and the burden of constant availability, possibly predisposing them for burnout.

#### Towards a better system?

The holistic midwives we interviewed for this article felt that they address a need that is not being met by the current maternity care system in the Netherlands. They made several suggestions for changes that they felt would improve matters. They believe the current maternity care system needs to address the root causes that drive women to holistic midwives and home births in high risk pregnancies: more continuity of caregiver, more room for a physiological approach and more flexibility in the use of protocols and guidelines. Currently, holistic midwives are addressing this need, because for some women, holistic midwives are the last resort before women feel they have no other option than to deliver unassisted. This situation is suboptimal for two reasons. First, many women do not actually desire a home birth with a holistic midwife; rather they feel driven to them. Following an initial traumatic experience, women often decide to take a more active role in the management plan for their next pregnancy and birth. However, they then encounter inflexible professionals, leading them to make a negative choice against the hospital, and instead choose a home birth in a high risk pregnancy [15,52]. Second, there is good reason for medical professionals to recommend a hospital birth in certain high risk situations like twins or breech births. As Bastian et al. found in Australia, mortality rates for home birth were 1:14 for breeches and 1:7 for twins. However, the authors lay responsibility for these poor outcomes squarely at the door of maternity care providers by stating that: “Overintervention and lack of choice for women with high risk pregnancies (…) could well encourage some to choose home rather than hospital birth” [53].

Preventing traumatic childbirth experiences could prevent situations in which women have lost all confidence in regular maternity care. In order to achieve this, holistic midwives feel that true client-centered maternity care needs to become the norm. This means that the protocol or guideline is the starting point of the conversation, instead of the bottom line. This does not mean that clients are allowed to dictate whatever treatment they feel is best for them; rather, that client and provider explore the possible options together, with more emphasis on client preference and personal situation and less strict adherence to protocols.

Another need that holistic midwives feel has to be addressed by regular maternity care is women’s need for more time and continuity of carer. For some women, these are prerequisites for developing a trusting relationship. And as de Vries states, “If fear is not balanced with trust, women are driven to make unwise choices” [54]. However, as Thompson [55] found, if trust is there, women will often stay in the system. In addition, a model where care is coordinated by (a team of) midwives, in close partnership with obstetricians when indicated in case of a high risk pregnancy leads to both increased satisfaction on the part of the client and yields better obstetrical outcomes such as more spontaneous birth, less pain medication and less preterm birth [3].

However, regardless of all points raised above, it is likely that some women will always seek out the services of holistic midwives, since, as Shorten and Shorten state, “The question is whether some women who employ independent midwives will ever be able to find what they need within mainstream services” [56].

### Strengths and limitations

#### Strengths

There are several strengths to this study. First, all authors have a background in maternity care and are very familiar with the Dutch system. This familiarity is reflected in the topic list. Second, for a qualitative study, it is extensive, with 24 midwives encompassing nearly all known holistic midwives in The Netherlands. Participants came from all parts of the country, all age groups and differing levels of work experience. Third, it is the first study examining the phenomenon of holistic midwifery in the Netherlands, a country known for its high percentage of home births, with community midwifery being an integral part of regular maternity care. Fourth, it is part of the larger WONDERstudy project, from which two literature studies, two qualitative studies and a survey have already been published. This allowed the interviewers to triangulate the themes from this study with those found in the previous interview study, as well as those known from the literature, which heightened the validity of the results of the current study.

#### Limitations

There are also some limitations to the current study. First, the fact that all authors are or have been part of the regular maternity care system may have motivated some participants to downplay their own involvement in their clients’ choices. It is possible that some midwives not only provided care as a last resort, but may have actually encouraged their clients to have a home birth in a high risk pregnancy. Although this question was asked, it was denied by the participants, but conflicting information on this subject can be easily found on the (Dutch) internet [41–43]. On the other hand, participants may have felt more at liberty to be honest with the interviewers, since we were known to be open to their points of view. Most of the themes mentioned by the participants correspond very well with those found by the same authors in their study on women’s motivations to give birth outside guidelines [15]. Second, one could suggest that the findings of this study cannot be extrapolated to other countries, since the Netherlands has a unique system of maternity care in which home birth in case of low risk pregnancy is an integral part of regular maternity care. On the other hand, the themes found in this study are not very different from those found in studies examining holistic or “independent” midwifery elsewhere. Finally, the sampling method could be seen as a limitation. However, according to many of the participants, the number of midwives interviewed closely approximates the total number of holistic midwives active in the Netherlands at that time, so it seems unlikely that many major themes were missed.

## Conclusions

This qualitative study analyzed holistic midwives’ motivations and approachto their work. Holistic midwives are a relatively small and new group of maternity care providers in the Netherlands, a country known for its physiological approach to childbirth and its integrated midwifery care. Four major themes were found: 1) The regular system is failing women, 2) The relationship as basis for empowerment, 3) Delivering client- centered care in the current system is demanding, and 4) Future directions. These themes all came together in one overarching theme which was “Addressing a need”.

Holistic midwives deliver an important service. They provide continuity of care and establish a relationship with their clients built on trust and mutual respect, truly putting their clients’ needs first. The type of care they deliver is actually that which both Dutch professional organizations encourage their respective members to provide. Holistic midwives may be the last resort before women choose to deliver unattended by any medical professional. In order to reduce women’s negative choices, which may place them and their unborn children at increased risk of a bad outcome, regular maternity care providers should focus on preventing traumatic childbirth experiences, while at the same time learning how to deliver second best care (in the eyes of the provider), so that no women will feel that regular care is no longer an option for them. In addition, more focus needs to be placed on continuity of caregiver, and a midwife-led continuity of carer model should be explored.

Some women will always prefer the care of holistic midwives, but currently, many of those who do feel that they have no other choice.
